# Eight-point lung ultrasonography protocol for assessing hypervolemia in chronic hemodialysis patients: A pilot study 

**DOI:** 10.5414/CNP104S04

**Published:** 2025-11-28

**Authors:** Luka Varda, Nejc Piko, Renata Smogavec, Nino Vreča, Sebastjan Bevc, Robert Ekart

**Affiliations:** 1 Department of Dialysis,; 2 Department of Nephrology, Clinic for Internal Medicine, University Medical Centre Maribor, and; 3 Faculty of Medicine, University of Maribor, Maribor, Slovenia

**Keywords:** lung ultrasound, hypervolemia, hemodialysis, arterial stiffness, bioelectrical impedance analysis

## Abstract

Background: Hypervolemia (HV) and arterial stiffness present an important problem for chronic hemodialysis (HD) patients. The most promising methods for evaluating excess fluid are bioelectrical impedance analysis (BIA) and lung ultrasonography with B-line assessment (LUS). The latter is traditionally performed in 28 anatomical locations on the front side of the chest. The study aimed to investigate whether a shorter LUS procedure in 8 locations correlates with other markers of HV and arterial stiffness. Materials and methods: We performed a single dialysis center observational study in adult chronic HD patients. Patients had to be without active malignancy, infection, chronic atrial fibrillation, carotid stenosis, severe aortic stenosis, or peripheral artery disease. We performed predialysis blood pressure measurements, LUS on 8 predefined locations, BIA, carotid-femoral pulse wave velocity (cfPWV) assessment, and laboratory values of the N-terminal prohormone of brain natriuretic peptide. Results: 19 patients were included, 7 male (36.8%). The median age of the patients was 71 years (IQR (60 – 74)), the median dialysis vintage was 51 months (IQR (27 – 87)). We found a statistically significant positive correlation between LUS and overhydration measured by BIA (r_s_ = 0.697; p < 0.001), LUS and intracellular water measured by BIA (r_s_ = 0.478; p = 0.038), and between LUS and extracellular water measured by BIA (r_s_ = 0.462; p = 0.046). Furthermore, we also found a statistically significant negative correlation between LUS and cfPWV (r_s_ = –0.539; p = 0.026). Conclusion: LUS in 8 locations is associated with markers of HV in HD patients, correlating positively with BIA measurements. Its correlation with cfPWV should be further investigated.

## Introduction 

End-stage kidney disease (ESKD) patients are at high risk of developing cardiovascular complications. The underlying pathophysiology is complex – anemia, vascular calcifications, uremic toxins, oxidative stress, and chronic inflammation contribute to the outcome [1]. Furthermore, arterial stiffness and hypervolemia have been recognized as significant risk factors for morbidity and mortality in this group of patients [2, 3]. 

Carotid-femoral pulse wave velocity (cfPWV) is an essential marker of central arterial stiffness and one of the most important predictors of cardiovascular health in ESKD patients. Changes in collagen, calcifications, and extracellular matrix content in the vascular wall are the main contributors to changes in PWV. It is also blood pressure (BP)-dependent, as higher BP causes stiffening of the vessels. The procedure is easy to perform, non-invasive, robust, and reproducible, making it one of the most commonly used markers for assessment of arterial stiffness [4]. 

Hypervolemia (HV) in ESKD patients undergoing hemodialysis (HD) has been associated with arterial hypertension [5], left ventricular hypertrophy [6], and increased mortality [2]. However, determining the euvolemic state in this group of patients poses a challenge. A gold standard for determining the dry weight has not yet been determined. Clinical examination, with the assessment of peripheral edema, auscultatory lung crackles, weight gain, and neck vein distention, is regularly used but appears insufficient with the new emerging technologies. 

Bioimpedance analysis (BIA) presents a promising method for estimating a patient’s dry weight. In a direct current circuit, the current flow is solely influenced by the circuit’s resistance at a given voltage. However, an additional factor – reactance – becomes significant with an alternating current. This adds another metric to the measurement, enabling BIA to differentiate between various fluid compartments. Although subject to possible errors depending on the body size, shape, and localized fluid buildup, whole body (wrist-to-ankle) bioimpedance is the most widely used clinical application of BIA [7]. Dry-weight reduction, based on BIA measurements, has been shown to improve BP control and left ventricular mass [8] and survival in HD patients [9]. 

Serum values of the N-terminal prohormone of brain natriuretic peptide (NT-proBNP) are elevated in the hypervolemic state due to the cardiac stretch. Furthermore, angiotensin II, endothelin, catecholamines, and hypoxia have also been linked to elevated serum levels of NT-proBNP. The latter can, therefore, be used as a marker of cardiac distress; however, it cannot distinguish between the causative factors (hormonal, hypervolemic, or hypoxic) [10]. It has previously been used as an indirect marker of hypervolemia in HD patients and can predict cardiovascular and all-cause mortality in this population [11]. 

In recent years, lung ultrasonography (LUS) has gained momentum in the field of volume assessment, mainly due to its ability to detect interstitial lung water and asymptomatic lung congestion. Furthermore, the procedure is non-invasive, free of radiation, easy to use, and offers acceptable inter- and intra-operator reproducibility. In contrast to A-lines, which are horizontal lines parallel to the pleural line and present in healthy lungs, LUS evaluates B-lines, vertical sonographic artefacts that begin at the pleural line and extend to the bottom of the screen. These comet-like structures emerge as the interstitium and alveoli are filled with fluid [12]. In a comprehensive anterolateral chest scan, 28 intercostal spaces are evaluated – 16 on the right side (second, third, fourth and fifth intercostal space in the parasternal, midclavicular, anterior axillary line, and medial axillary line) and 12 on the left (second, third and fourth intercostal space in the parasternal, midclavicular, anterior axillary line, and medial axillary line) [13]. Because there is usually limited time to perform the exam in clinical practice, shorter LUS protocols have also been introduced [14]. This study aimed to determine how an 8-point LUS protocol correlates with BIA measurements, predialysis BP, serum levels of NT-proBNP, and non-invasive measurements of cfPWV in chronic HD patients. 

## Materials and methods 

### Study population and design 

We conducted an observational study in a single dialysis center – the University Medical Center Maribor. The study was conducted in accordance with the principles of the Declaration of Helsinki and was approved by the local review board (approval number: UKC-MB-KME-24-04/17). Because all the measurements performed in the study are also regularly used in our clinical practice, oral consent was obtained from all included patients. We included 19 randomly selected HD patients who met the inclusion criteria. A pilot study is presented; therefore, further inclusion of patients is ongoing. Patients had to be at least 18 years old, treated with HD for at least 3 months, without active infection or malignancy and hospitalization for any cause in the previous month. Patients had to be without any limb amputations. Because arterial stiffness measurements were performed, we also excluded patients with known chronic atrial fibrillation, carotid stenosis, severe aortic stenosis, and peripheral artery disease. The patient’s non-cooperation was considered an exclusion criterion. The ultrafiltration rate for all patients in the previous HD session was set to achieve the patient’s dry weight. We performed predialysis BP measurements using a standardized method, LUS on 8 predefined locations, and assessment of cfPWV. All measurements for a single patient were performed on the same day before the mid-week dialysis session. Blood was also drawn before the mid-week dialysis session to obtain serum NT-proBNP values. 

### Lung ultrasonography 

B-lines were assessed with LUS (VScan, General Electrics Corporate, Horten, Norway). A cardiac probe with a frequency range between 2.5 and 7.5 MHz was used. B-lines were assessed on 8 predefined anatomical locations on the front side of the chest – 4 locations on each side (second and fourth intercostal space in the parasternal line and anterior axillary line). The protocol was adjusted based on pre-existing 8-point protocols [14, 15]. B-lines were counted in each intercostal space. If a confluence of B-lines was present, we estimated the percentage of intercostal space covered with B-lines and divided it by 10. The described procedure enabled a quantitative or semiquantitative estimation of interstitial lung water. 

### Bioelectrical impedance analysis 

BIA measurement was performed using a Body Composition Monitor (BCM, Fresenius Medical Care, Bad Homburg, Germany). In a supine position, 4 electrodes were adjusted to the patient – 2 on the dorsal side of the wrist and 2 on the anterior surface of the ankle on the same body side (non-fistula or non-catheter side). Before the beginning of the measurement, the patient’s gender, age, height, weight, and systolic and diastolic BP measurements had to be entered into the software. The system uses the bioimpedance spectroscopy technique. Total body water (TBW), intracellular water (ICW), and extracellular water (ECW) were calculated through the electrical conductance in a cell suspension [16]. A body composition model uses the information about ECW and TBW and calculates the three principal body components – overhydration (OH), lean tissue mass (LTM), and adipose tissue mass (ATM) [17]. 

### Carotid-femoral pulse wave velocity 

We performed non-invasive measurements of cfPWV (Sphygmocor Xcel, Atcor Medical, Sydney, Australia) before the mid-week dialysis session. Patients were put in a supine position. According to the manufacturer’s guidelines, the blood pressure cuff was adjusted to the patient’s thigh. The distance between the carotid artery’s pulse and the sternal notch and between the sternal notch and the top edge of the femoral cuff was measured in millimeters. The measurements were entered into the software alongside BP measurements and the patient’s height, gender, and date of birth. The system measured the PWV of the arterial pulse waveform travelling through the descending aorta to the femoral artery. The aortic PWV was detected from carotid and femoral pulses simultaneously. The carotid pulse was measured using a tonometer, while the femoral pulse was measured through pulsations in the pre-adjusted cuff on the patient’s thigh. When both signals were of sufficient quality, the waveform on the screen turned green. After 10 seconds of simultaneous valid signals, a report screen appeared with the calculation of PWV. 

### Statistical analysis 

All continuous variables are presented as mean value ± standard deviation, or median value (interquartile range (IQR)), depending on the data distribution, and all categorical variables as frequencies and percentages. Correlation analysis was performed using Pearson or Spearman’s correlation tests, depending on the data distribution. The data was analyzed using the commercially available SPSS® (version 29.0, Chicago, IL, USA). Statistical significance was set at p < 0.05. 

## Results 

The study included 19 adult chronic HD patients. Seven patients were male (36.8%). The median age of the patients was 71 years (IQR (60 – 74)), and the median dialysis vintage was 51 months (IQR (27 – 87)). 17 (89.5%) were dialyzed via arteriovenous fistula, the rest via central venous catheter, and all were treated with on-line postdilution hemodiafiltration. The causes of ESKD in the cohort were arterial hypertension in 4 patients (21.1%), diabetes mellitus in 5 patients (26.3%), glomerulonephritis in 6 patients (31.6%), polycystic kidney disease in 3 patients (15.8%), in 1 patient (5.3%) the cause was undetermined. 

We found a statistically significant positive correlation between B-lines on LUS and OH measured by BIA (r_s_ = 0.697; p < 0.001), B-lines on LUS and ICW measured by BIA (r_s_ = 0.478; p = 0.038), and B-lines on LUS and ECW measured by BIA (r_s_ = 0.462; p = 0.046). Furthermore, we also found a statistically significant negative correlation between B-lines and cfPWV (r_s_ = –0.539; p = 0.026). B-lines on LUS did not correlate with NT-proBNP values or systolic and diastolic BP measurements. Median values of B-lines on LUS, BIA, and BP measurements, NT-proBNP values, and cfPWV values are presented in Table 1. We did not find a statistically significant correlation between cfPWV, BP, BIA measurements, or NT-proBNP. NT-proBNP and systolic and diastolic BP measurements did not correlate with any of the volume markers used in the study or cfPWV. 

## Discussion 

In this study, we found a statistically significant positive correlation between LUS performed in 8 locations and OH, ECW, and ICW measured by BIA, which are most commonly used for evaluating volume overload. As presented in Table 1, the number of B-lines found on LUS and OH volume measured by BIA was relatively low. Thus, both measurements correlate well, even in patients with relatively well-controlled volume status, and they might be used interchangeably or together. This could be useful, especially in patients where BIA does not provide sufficient data. We must acknowledge that the correlation coefficient was high only in the correlation between LUS and OH (r_s_ = 0.697; p < 0.001), indicating a possible clinical significance, whereas other correlation coefficients were lower. 

The high cardiovascular mortality of HD patients is mostly attributed to hypertension and cardiac disease. Both are associated with fluid overload and arterial stiffness. In a randomized controlled trial, assessment of BIA provided better management of fluid status in patients on maintenance HD, which led to a significant reduction of left ventricular mass index, ambulatory BP measurements, and arterial stiffness compared to the control group where the ultrafiltration rate was not adjusted according to the measurements by BIA [8]. Therefore, BIA is considered one of the most promising methods for assessing fluid status in chronic HD patients. It is non-invasive, portable, safe, economical, and can also provide information about fat and lean tissue mass. However, it measures the body composition indirectly, meaning the accuracy depends on the underlying mathematical models, which have been validated only on certain cohorts, mostly without kidney disease [18]. 

Other methods for determining hypervolemia have been sought in the last decade. A comprehensive LUS protocol performed on 28 locations on the front side of the chest was a good foundation to guide the strategy for volume reduction in patients treated with HD with high cardiovascular risk [13]. The same protocol was also able to reduce ambulatory BP measurements [19]. LUS thus appears to be a suitable option for assessing volemia and determining the level of ultrafiltration in HD patients. However, in clinical practice, more than one method of determining overhydration is usually used to predict the ultrafiltration rate correctly. Therefore, shorter LUS protocols are being assessed. Our group previously showed a good correlation between 28- and 8-point LUS protocols in patients on maintenance HD [15]. Leidi et al. [20] conducted a prospective study comparing 28-point and 8-point protocols in acute heart failure patients. The latter had equal reproducibility and was performed and interpreted significantly quicker by novice and expert practitioners. The mean time difference in conducting the examination was 3.6 minutes for experts and 5.1 minutes for novices. Experts interpreted the results 6 minutes quicker and novices 6.3 minutes quicker [20]. Similarly, in chronic HD patients, the 8-point protocol was non-inferior to the 28-point protocol in detecting pulmonary congestion or the prediction of cardiovascular events and death over a 3-year observational period. Meanwhile, the 8-point protocol was performed 1.7 minutes faster on average [14]. With our analysis, we confirmed a good correlation between a short LUS protocol and volume markers measured by BIA. Shorter LUS protocols should be further evaluated in prospective clinical trials in larger cohorts to confirm their clinical utility. In case of clinical uncertainty, 28-point protocols might provide better insight. 

HV is an essential factor for hypertension in HD patients [5]. Our study did not find a significant correlation between LUS and predialysis BP measurements, nor between BIA and predialysis BP measurements. Hypertension in this group of patients is multifactorial and exhibits complex pathophysiology. While volume expansion is an essential element, other factors should also be considered. Additionally, our patients were not severely hypervolemic (the median OH measured by BIA was only 0.8 L, and the median number of B-lines was 7), which may have influenced the outcome. This is likely the primary reason we did not identify a statistically significant association between volume markers and NT-proBNP levels. Future studies should concentrate on ambulatory BP measurements as they remain the gold standard for diagnosis and treatment decisions in HD patients. 

We found a statistically significant negative correlation between LUS and cfPWV. This result is unexpected because some studies found a positive correlation between arterial stiffness and volume overload [21]. However, cfPWV did not correlate with BIA, BP measurements, or NT-proBNP. As mentioned before, our group of patients were not highly hypervolemic and their cfPWV was within the normal range (median value 7.5 m/s (IQR (6.9 – 8.0)), which is not representative of the average HD cohort. Although cfPWV can, in some cases, be influenced by acute hemodynamic changes, such as arterial hypertension [4], arterial stiffening is generally a chronic process. Thus, it is possible that in patients with normal values of cfPWV (indicating that vascular changes are not yet severe), the correlation with B-lines can also be negative. This association should be evaluated further on a larger cohort of HD patients with similar characteristics or in patients more representative of the HD population. 

Our study has several limitations. It was performed in a single dialysis center, and inclusion and exclusion criteria were strictly defined. Therefore, the sample size is small. The patients were not severely overhydrated, which could have influenced the results. Although patients were randomly selected, this does not accurately reflect the actual condition of many HD patients. Furthermore, the analysis is based on a single predialysis measurement of each parameter. However, our results may inspire further research in this area, primarily to determine the association between markers of arterial stiffness and short LUS protocols in HD patients. Future research should aim for external validation of results in independent cohorts of patients and multi-center randomized clinical trials in larger cohorts. 

In conclusion, LUS in 8 locations on the front side of the chest is associated with HV markers in HD patients. In this observational study, it correlated positively with volume markers measured by BIA and negatively with cfPWV but not with predialysis BP measurements and NT-proBNP. 

## Compliance with ethical standards 

The study was performed according to the principles of the Declaration of Helsinki. It has been approved by the local review board (approval number: UKC-MB-KME-24-04/17). 

## Authors’ contributions 

L.V., N.P., and R.E. designed the study. L.V., R.S., and N.V. performed all measurements in the study. L.V. collected all data, performed the statistical analysis, and wrote the manuscript. L.V., N.P., R.S., N.V., S.B., and R.E. read and critically evaluated the manuscript and gave final approval for publication. 

## Funding 

The authors did not receive grants or other funding for this work. 

## Conflict of interest 

The authors have no conflict of interest to declare. 

**Table 1.** Median values of counted B-lines in lung ultrasound, parameters measured by bioelectrical impedance analysis, blood pressure measurements, and laboratory values of N-terminal prohormone of brain natriuretic peptide with correlation coefficients and p-values.

Parameter 1 (median (IQR))	Parameter 2 (median (IQR))	Pearson/Spearman’s correlation coefficient (p-value)
LUS (8 locations) 7 B-lines (4 – 14)	Overhydration (BIA) 0.8 L (0.3 – 2.9)	**0.697 (< 0.001)**
	Intracellular water (BIA) 17.2 L (14.9 – 20.8)	**0.478 (0.038)**
	Extracellular water (BIA) 15.8 L (14.9 – 21.1)	**0.462 (0.046)**
	cfPWV 7.5 m/s (6.9 – 8.0)	**–0.539 (0.026)**
	NT-proBNP 582 (210 – 1,092)	0.142 (0.562)
	Systolic blood pressure 143 mmHg (129 – 146)	0.070 (0.776)
	Diastolic blood pressure 78 mmHg (67 – 92)	0.337 (0.159)

LUS = lung ultrasound; BIA = bioelectrical impedance analysis; cfPWV = carotid-femoral pulse wave velocity; NT-proBNP = N-terminal prohormone of brain natriuretic peptide; CI = confidence interval. Bold = statistically significant.
